# Ferritinophagy-Mediated ROS Production Contributed to Proliferation Inhibition, Apoptosis, and Ferroptosis Induction in Action of Mechanism of 2-Pyridylhydrazone Dithiocarbamate Acetate

**DOI:** 10.1155/2021/5594059

**Published:** 2021-10-14

**Authors:** Longlong Li, Hao Li, Yongli Li, Jiankang Feng, Deng Guan, Yalei Zhang, Yun Fu, Shaoshan Li, Changzheng Li

**Affiliations:** ^1^Department of Surgery, The Third Affiliated Hospital of Xinxiang Medical University, Xinxiang, Henan 453003, China; ^2^Department of Histology and Embryology, Sanquan College of Xinxiang Medical University, Xinxiang, Henan 453003, China; ^3^College of Basic Medical Science, Xinxiang Medical University, Xinxiang, Henan 453003, China; ^4^College of Pharmacy, Sanquan College of Xinxiang Medical University, Xinxiang, Henan 453003, China

## Abstract

Reactive oxygen species (ROS) production is involved in the mechanism of action of a number of drugs, but the biological effects of ROS remain to be clarified. Furthermore, ferroptosis involves iron-dependent ROS production that may be derived from ferritinophagy; however, the association between ferroptosis and ferritinophagy has not been fully established. The present study demonstrated that dithiocarbamate derivatives (iron chelators) exhibited antineoplastic properties involving ferritinophagy induction, but whether the underlying mechanisms involved ferroptosis was unknown. To gain insight into the underlying mechanism, a dithiocarbamate derivative, 2-pyridylhydrazone dithiocarbamate s-acetic acid (PdtaA), was prepared. An MTT assay demonstrated that PdtaA inhibited proliferation involving ROS production (IC_50_ = 23.0 ± 1.5 *μ*M for HepG2 cells). A preliminary mechanistic study revealed that PdtaA induced both apoptosis and cell cycle arrest. Notably, PdtaA also induced ferroptosis via downregulation of GPx4 and xCT, which was first reported for a dithiocarbamate derivative. Moreover, these cellular events were associated with ROS production. To explore the origin of ROS, expression of the ferritinophagy-related genes, ferritin, and nuclear receptor coactivator (NCOA4) were measured. Immunofluorescence and western blotting analysis indicated that PdtaA-induced ferritinophagy may contribute to ROS production. To investigate the role of ferritinophagy, autophagy inhibitor 3-methyladenin or genetic knockdown of NCOA4 was employed to inhibit ferritinophagy, which significantly neutralized the action of PdtaA in both apoptosis and ferroptosis. Taken together, PdtaA-induced cell cycle arrest, apoptosis, and ferroptosis were associated with ferritinophagy.

## 1. Introduction

Liver cancer is the third leading cause of deaths from cancer worldwide [1]. Chemotherapy is still an important clinical treatment, but its side effects and drug resistance make its therapeutic effect limited [2]. Recently, the regorafenib has been approved for treatment of advanced hepatocellular carcinoma; however, the treatment for liver cancer is not ideal; clearly, an effective therapeutic strategy still is required [3]. Thus, the studies should be focused on new molecularly targeted agents and assessing the benefits of combined molecular therapy under the heterogeneity condition of tumor [4]. In addition, the tumor microenvironment serves an important role in tumor metastasis [5], in particular metalloproteinases and cytokines degrade the extracellular matrix and recruit and suppress incoming immunocytes (such as monocyte), respectively. The recruited monocytes were differentiated and tamed to tumor-associated macrophages, which can phagocytize apoptotic or necrotic cells, as well as senescent blood cells, supplying the necessary iron, copper ions, and cytokines for tumor cell proliferation and angiogenesis [6, 7]. Therefore, depletion of the metal ion by chelation may be another option for cancer therapy.

Reactive oxygen species (ROS) are radicals, ions, or molecules that possess a single unpaired electron [8]. Mitochondria are the main site for ROS production [9]. In addition, ROS are also generated by the degradation of iron-containing macromolecules (ferritin and mitochondrial components) or endocytosed erythrocytes (by macrophages) in lysosomes. The released iron triggers the Fenton reaction, yielding reactive hydroxyl radicals [10]. Ferritinophagy is a nuclear receptor coactivator- (NCOA4-) mediated process of ferritin degradation. A few of iron chelators can induce ferritinophagy, but whether ROS production is involved seems to depend on characteristic of iron chelator. For example, deferoxamine (DFO) is a ferritinophagy inducer but is used as ROS scavenger [11], while dithiocarbamate derivatives function as both ferritinophagy and ROS inducers [12, 13]. In general, most chemotherapeutic agents exerted their action involved ROS production [14, 15], which resulted in proliferation inhibition (downregulation of Ki-67) [16], apoptosis (characterized by nuclear fixation, cell shrinkage, DNA fragmentation, and cell membrane bubbles) [17–19] and cell cycle delay [20, 21]. There are two pathways of apoptosis induction: the extrinsic and intrinsic pathways [22].

Ferroptosis is a type of iron-dependent cell death and is distinct from apoptosis, although ROS production has a role in both. In ferroptosis, cell death is induced by ROS-mediated peroxidation of polyunsaturated fatty acids [23, 24]. Lipid peroxidation can be decreased by glutathione (GSH). Cysteine is a material for GSH synthesis, derived from cystine reduction. Cystine is transported into the cell through the system Xc- transporter and the solute carrier family 7 member 11 (SLC7A11) subunit. Therefore, the glutathione peroxidase 4 (GPx4)/Xc- system modulates cellular redox homeostasis and determines ferroptosis fate [25, 26]. In addition, ferroptosis was also a type of autophagy-dependent cell death [27].

Dithiocarbamate is an important iron chelator, which is used in the treatment of bacterial and fungal infection, acquired immunodeficiency syndrome, and human cancer [28]. In view of this, numerous new dithiocarbamate derivatives have been investigated, including a series of pyridylaldehyde (ketone) dithiocarbamate hydrazones prepared in our laboratory, which have good antitumor activity. A preliminary mechanism study showed that dithiocarbamate derivatives can be used as nuclear factor *κ* B inducers [29], proteasome inhibitors [30], and DNA intercalators [31]. However, whether there is an association between ferritinophagy and ferroptosis has not been fully established [32–34]. The current study investigated the mechanism of action of 2-pyridylhydrazone dithiocarbamate s-acetic acid (PdtaA) that prepared by a three-step reaction in HepG2 cells [35]. To the best of our knowledge, the present study is the first to report the dithiocarbamate-mediated induction of ferroptosis, which will improve our knowledge of ferroptosis.

## 2. Results

### 2.1. PdtaA Induces Proliferation Inhibition and Cell Cycle Arrest Is ROS Dependent

**Figure 1:**
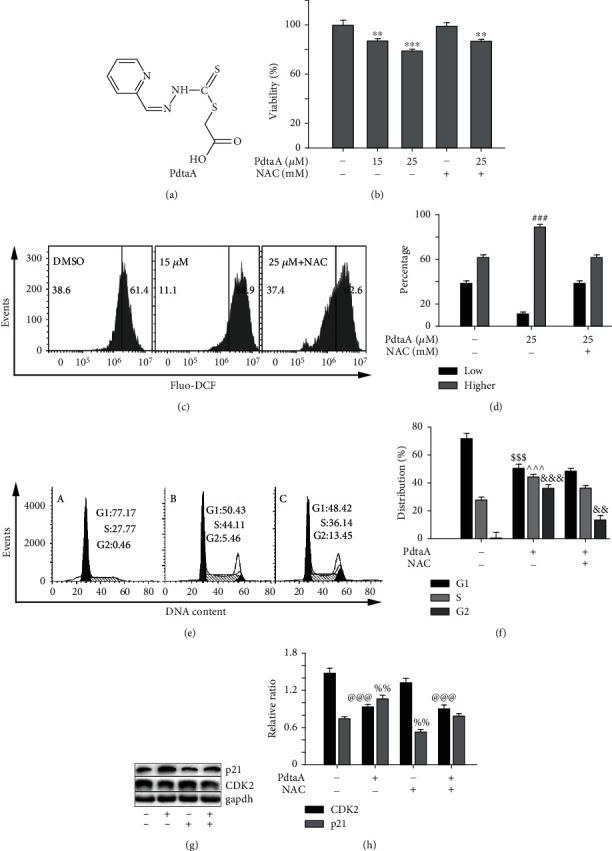
PdtaA-induced proliferation inhibition and cell cycle arrest involves ROS production. (a) Structure of PdtaA. (b) Proliferation inhibition induced by PdtaA. (c) PdtaA-induced ROS production. (d) Quantification from (c). (e) PdtaA-induced S phase arrest: (a) Control. (b) PdtaA. (c) PdtaA plus NAC. (f) Quantification analysis from (e). (g) Western blotting analysis of cell cycle-related proteins. (h) Quantification from (g). ^∗∗^^,&&,%%^*P* < 0.05, ^∗∗∗^^,###,$$$,^^^,&&&,@@@^*P* < 0.01 vs. the control.

PdtaA was prepared by a three-step reaction from our laboratory. The new compound was purified by flash chromatography, and the structural characteristic of PdtaA was determined by NMR and HRMS spectra (Figure 1(a)). Next, the effect of PdtaA on proliferation inhibition against the HepG2 cell was preliminarily evaluated. The antiproliferative activity of the agent at given concentrations is depicted in Figure 1(b). Notably, 15 and 25 *μ*M PdtaA displayed significant proliferation inhibition for 24 h (*P* < 0.01 and *P* < 0.001, respectively). In addition, chemotherapeutic agents often involve ROS production in a mechanism; PdtaA-induced proliferation inhibition might have similar mechanisms. Thus, the cellular ROS content after PdtaA treatment was further measured using flow cytometry. As shown in Figure 1(c), the populations in higher fluorescence intensities significantly increased by ~27% in PdtaA-treated cells compared with that of the control; however, the addition of ROS scavenger, N-acetyl-L-cysteine (NAC), could significantly attenuate ROS production (~15%) (Figures 1(c) and 1(d)), indicating that the action of PdtaA involved ROS production. However, whether ROS production contributed to proliferation inhibition remained to be further determined. To this end, proliferation inhibition in the presence of NAC was further assessed. As showed in Figure 1(b), addition of NAC (1.5 mM) could attenuate the inhibitory ability of PdtaA, but not fully neutralized; this indicated that ROS production partly contributed to the proliferation inhibition. In addition, Ki-67 is known to be a cell proliferation marker; the growth inhibition induced by the agent prompted us to further determine the alteration of it. As shown in Fig. S1, PdtaA treatment resulted in the depletion of Ki-67, indicating that the proliferation inhibition correlated with downregulation of Ki-67 (Figure 1(b)) based on the western blotting analysis.

In addition, Ki-67 also has role in cell cycle regulation; the depletion of Ki-67 may disturb cell cycle of the HepG2 cells; thus, the cellular DNA content was determined by flow cytometry after the cells were stained with propidium iodide. As shown in Figure 1(e), PdtaA treatment caused a significant increase in S phase proportions (the percentage at S phase was increased from 27.72 to ~44% (Figures 1(b)–1(e)) when the cells were exposed to PdtaA for 24 h. However, addition of NAC could significantly attenuate, but not fully rescue the proportions in S phase (Figures 1(c)–1(e)), indicating that the cell cycle delay partly depended on ROS production. The quantification analysis in populations at different phases is presented in Figure 1(f). To resolve the molecular basis of cell cycle delay, the expression changes of cell cycle-related proteins were determined. Western blotting analysis revealed that the level of cyclin-dependent kinase 2 (CDK2) was downregulated, whereas p21 was upregulated after PdtaA treatment (Figure 1(g)). These differences were statistically significant (Figure 1(h)). Similarly, addition of NAC could significantly attenuate, but not fully rescue these PdtaA-mediated changes, such as CDK2, suggesting that the notion that PdtaA-induced cell cycle arrest was partly ROS dependent. Further study revealed that CDK2 downregulation was associated with enhanced ferritinophagy (Fig. S2).

### 2.2. PdtaA Induces Apoptosis

**Figure 2:**
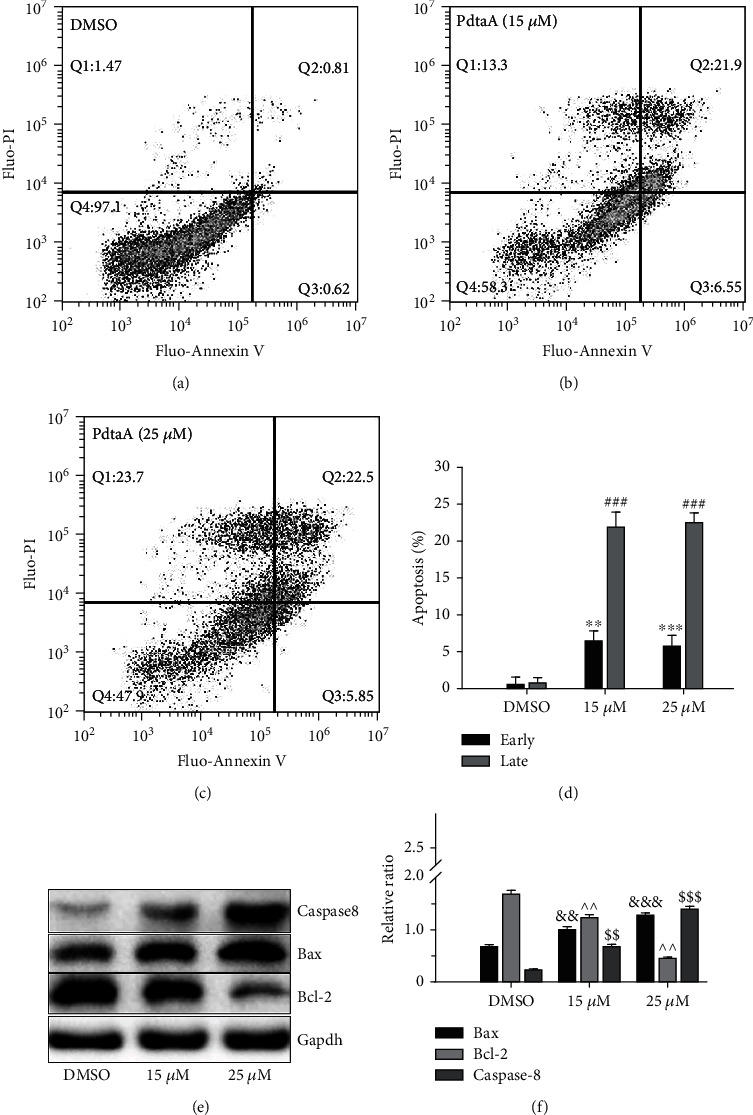
PdtaA induces apoptosis. Apoptosis analysis: (a) 70% DMSO, (b) 15 *μ*M PdtaA, and (c) 25 *μ*M PdtaA. (d) Quantification of flow cytometry analyses. ^∗∗^*P* < 0.05 vs. the control; ^∗∗∗^^, ###^*P* < 0.01 vs. the control. (e) Immunoblotting analysis of apoptosis-related proteins and (f) quantification. ^&&,^^,$$^*P* < 0.05 vs. the control; ^&&&,$$$^*P* < 0.01 vs. the control.

The presence of phosphatidylserine in the outer leaflet of the plasma membrane is a surface change common to apoptotic cells; thus, Annexin V with high affinity for phosphatidylserine was used to detect the phosphatidylserine. The flow cytometry analysis indicated that PdtaA primarily induced early and late apoptosis in a concentration-dependent manner (Figures 2(a)–2(c), from 1.4 to 28.4%). The differences between the control and PdtaA-treated cells were significant (*P* < 0.01 or *P* < 0.001; Figure 2(d)). In addition, concomitant to the apoptosis, notable necrosis was also observed (Figures 2(a)–2(c)). To further support the aforementioned conclusion, the levels of apoptosis-related proteins were also measured. Western blotting analysis revealed that the proapoptotic BCL2 (BCL2 apoptosis regulator) associated x (Bax) was increased compared with that of the control (Figure 2(e)), and contrarily the prosurvival protein Bcl-2 was downregulated, which was indicative of involvement of apoptosis in PdtaA action. The quantification analysis is presented in Figure 2(f), with a significant difference between the control and PdtaA-treated groups (*P* < 0.05). Next, the causes of apoptosis were further analyzed. We speculated that PdtaA-induced apoptosis may be from ROS production. To this end, a ROS scavenger (NAC) was used to test the hypothesis. As shown in Fig. S3, addition of NAC indeed attenuated the ability of PdtaA in apoptosis induction, supporting that the apoptosis induction involved ROS production. Similarly, an autophagy inhibitor (3-MA) also decreased the proportion of apoptosis induced by PdtaA. On the other hand, the addition of NAC did not fully neutralize (rescue) the apoptosis induction, implying that ROS production plays a partial role in the mechanism of action of PdtaA.

### 2.3. PdtaA Induces Ferroptosis

**Figure 3:**
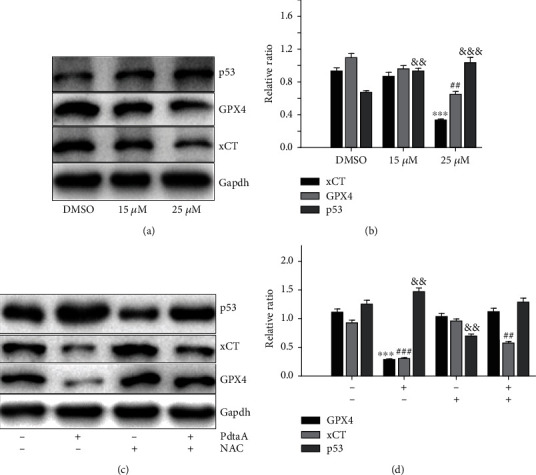
PdtaA induces ferroptosis. (a) Western blotting analysis of ferroptosis-related proteins and (b) quantification. (c) NAC could attenuate the action of PdtaA on regulation of ferroptosis-related proteins and (d) quantification. ^∗∗^^,##,&&^*P* < 0.05, ^∗∗∗^^,###,&&&^*P* < 0.01 vs. the control.

Ferroptosis is an iron-dependent accumulation of ROS that leads to lipid peroxidation and cell death. Inhibition of GPx4 activity leads to the accumulation of lipid peroxidation that is a significant hallmark and indicator of ferroptosis. Since PdtaA induced ROS production in the present study, it was hypothesized that ferroptosis might occur. To this end, the level of ferroptosis-related proteins, such as GPx4, xCT, and p53, before and after PdtaA treatment was determined using immunoblotting. As shown in Figure 3(a), PdtaA could downregulate both GPx and xCT in a concentration-dependent manner, but the level of p53 was upregulated (Figure 3(a)), suggesting that PdtaA induced ferroptosis was through inhibiting the xC- system and GSH synthesis. p53 also played a role in ferroptosis induction. The quantification analysis of these genes is shown in Figure 3(b), demonstrating significant protein level changes between the control and PdtaA-treated cells (at higher concentration, *P* < 0.01 or *P* < 0.001). To confirm that the occurrence of ferroptosis was associated with ROS production, the ROS scavenger, NAC, was added during the PdtaA treatment. As shown in Figure 3(c), NAC significantly attenuated the regulatory effect of PdtaA on ferroptosis-related proteins. The quantification analysis is presented in Figure 3(d).

In addition, the changes in lipid peroxidation and GSH content were further investigated. As shown in Fig. S4A, the abundance of GSH was significantly decreased with PdtaA treatment, in accordance with increase of ROS production (Figure 1). In addition, 3-MA markedly attenuated the action of PdtaA, indicating that autophagy was involved in the process. Similarly, inhibition of p53 also attenuated the action of PdtaA. Since GSH decreased, lipid peroxidation was expected to be increased. To this end, lipid peroxidation was further determined. As shown in Fig. S4B, the abundance of lipid peroxidation significantly increased, associated with decreased reduced GSH after PdtaA treatment. In addition, ferrostatin-1 (ferroptosis inhibitor) could partly neutralize the action of PdtaA, suggesting that ferroptosis was involved in action of PdtaA. Similar results from western blotting analysis also supported this conclusion, because addition of ferrostatin-1 attenuated markedly the action of PdtaA on regulation of xCT, GPx4, and p53, confirming that PdtaA indeed induced ferroptosis (Fig. S5).

### 2.4. ROS Production Is Partly due to Occurrence of Ferritinophagy

**Figure 4:**
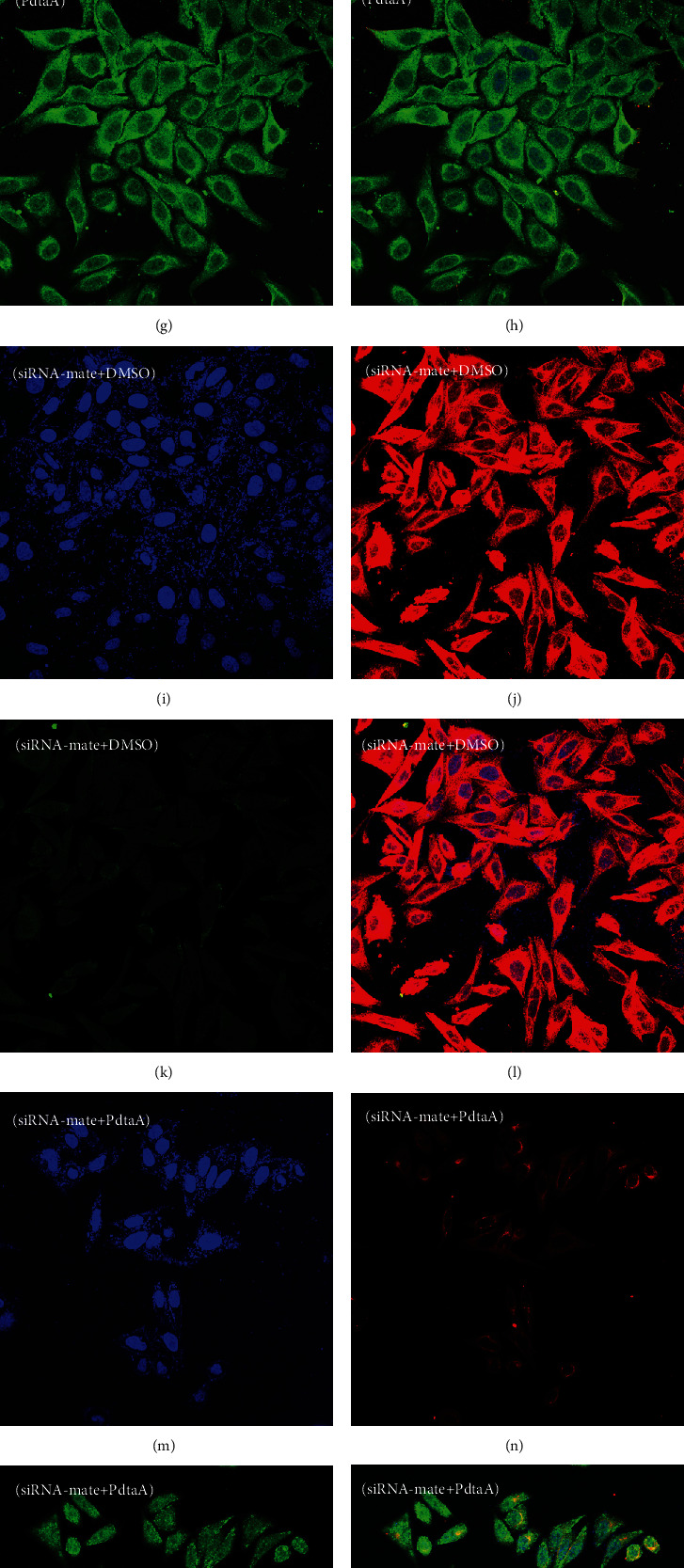
PdtaA induces ferritinophagy. Nuclei stained by DAPI in blue, ferritin labeled in red, and NCOA4 labeled in green. Red or green fluorescence is due to the secondary antibody attached different fluorescence groups; at given excitation wavelength, the emission of the groups showed either red or green. (a–d) Control (70% DMSO): (a) nuclei, (b) ferritin, (c) NCOA4, and (d) merge of ferritin with NCOA4. (e–h) The PdtaA-treated group: (e) nuclei, (f) ferritin, (g) NCOA4, and (h) merge of ferritin with NCOA4. (i–l) siRNA-mate: (i) nuclei, (j) ferritin, (k) NCOA4, and (l) merge of ferritin with NCOA4. (m–p) PdtaA plus siRNA-mate: (m) nuclei, (n) ferritin, (o) NCOA4, and (p) merge of ferritin with NCOA4. (q–t) PdtaA plus siRNA-NCOA4: (q) nuclei, (r) ferritin, (s) NCOA4, and (t) merge of ferritin with NCOA4. Scale bar, 100 *μ*m.

As ROS production was involved in the action of PdtaA, the potential source of ROS was investigated. Previous studies revealed that dithiocarbamate derivatives could induce ferritinophagy that partly contributed to ROS production; therefore, PdtaA might have a similar function. To this end, the alterations of cellular ferritin and NCOA4, a specific carrier for ferritin degradation, were measured via immunofluorescence. As shown in Figure 4, PdtaA treatment resulted in a decrease of red fluorescence of ferritin (Figure 4(f)) compared with the control (Figure 4(b)). On the contrary, green fluorescence of NCOA4 (Figure 4(g)) was increased compared with the control (Figure 4(c)). The merged images in Figures 4(d) and 4(h) show before and after PdtaA treatment, respectively. Those changes hinted that the ferritin degradation might occur through autophagic proteolysis, or ferritinophagy induction. To corroborate the role of NCOA4 in ferritinophagy, NCOA4 was knocked down using siRNA (Fig. S6). Comparing the level of NCOA4 in PdtaA-treated cells (Figure 4(o)) with that of siRNA-mate (Figure 4(k)), NCOA4 was significantly upregulated, and ferritin was downregulated. However, the regulatory effect of PdtaA on ferritin was almost neutralized (Figures 4(r) and 4(j)) when NCOA4 was knocked down, similar situation for NCOA4 (Figures 4(s) and 4(k)). Therefore, the alterations in ferritin and NCOA4 under those conditions suggested that ferritinophagy occurred.

**Figure 5:**
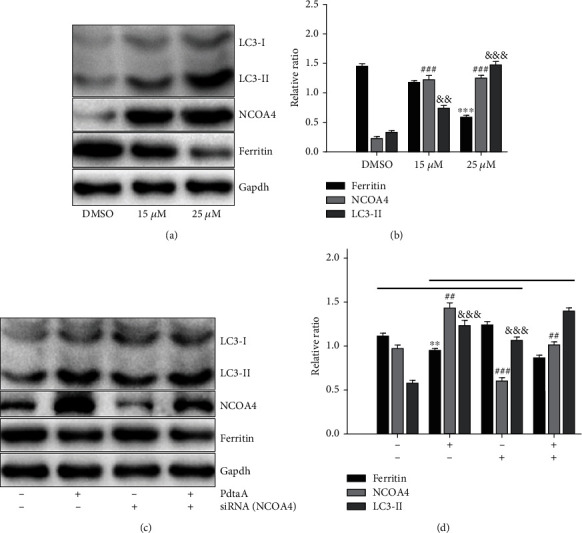
PdtaA induces ferritinophagy. (a) The effect of PdtaA on autophagy-related proteins and (b) quantification. (c) Knockdown of NCOA4 attenuated the action of PdtaA in ferritinophagy induction and (d) quantification. ^∗∗^^,##,&&^*P* < 0.05, ^∗∗∗^^,###,&&&^*P* < 0.01 vs. the control.

To further validate this, immunoblotting analysis was also conducted.  Figure 5 showed that PdtaA treatment resulted in a downregulation of ferritin and upregulation for NCOA4 and LC3-II, further demonstrating that PdtaA induced ferritinophagy. This was in accordance with the immunofluorescence results. The quantification analysis is presented in Figure 5(b). The alteration in ferritin, LC3-II, and NCOA4 after PdtaA treatment was significant (*P* < 0.05). Similarly, knockdown of NCOA4 markedly attenuated the action of PdtaA on regulation of levels of ferritin and NCOA4 compared with PdtaA only; however, knockdown of NCOA4 did not fully neutralize the regulatory effect of PdtaA on NCOA4. This was due to the unequal regulatory power between PdtaA and siRNA. In fact, the regulatory effect of both siRNA and PdtaA were a concentration-dependent manner. The increase of NCOA4 protein levels even the presence of siRNA-NCOA4 was due to higher regulatory power of PdtaA than that of siRNA (Figure 5(c)), a lower concentration of the agent used may achieve the balance. The quantitative comparisons of LC3, ferritin, and NCOA4 are shown in Figure 5(d), suggesting that NCOA4 played an important role in ferritin degradation. In addition, the total iron content that represents ferritin abundance at the indicated conditions was further determined using atomic absorption spectra. As shown in Fig. S7, PdtaA resulted in decreased total iron abundance, while knockdown of NCOA4 led to increased iron abundance.

### 2.5. Apoptosis Is Associated with Ferritinophagy

**Figure 6:**
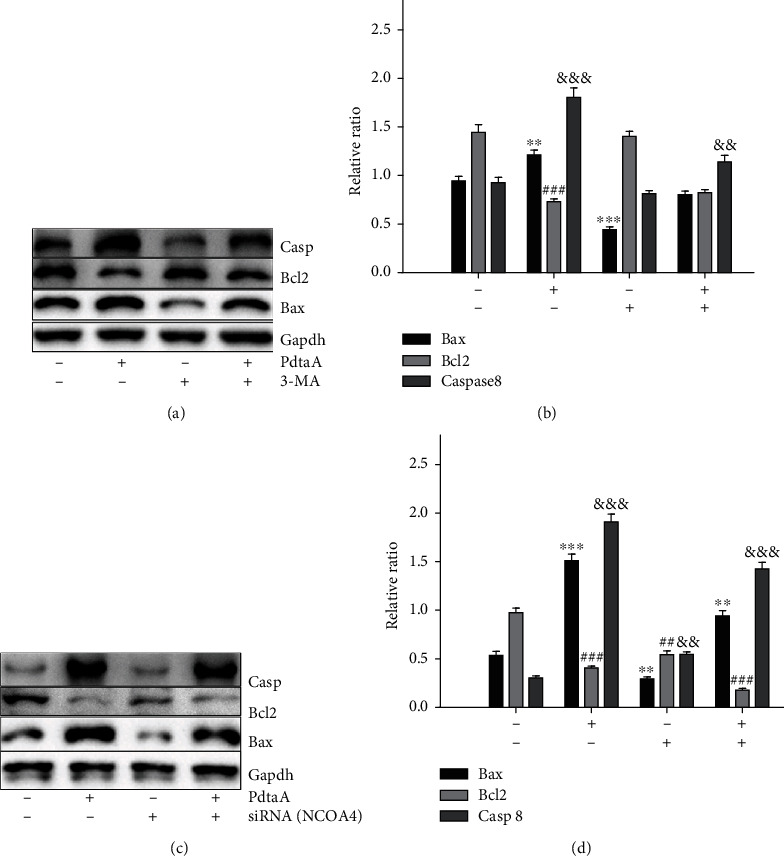
PdtaA induces apoptosis associated with ferritinophagy. (a) 3-Methyladenin attenuated the effect of PdtaA on apoptosis regulation and (b) quantification. (c) Knockdown of NCOA4 counteracted the ability of PdtaA for apoptosis induction and (d) quantification. ^∗∗^^,##,&&&^*P* < 0.05, ^∗∗∗^^,&&&,###^*P* < 0.01 vs. the control.

As aforementioned, PdtaA-induced apoptosis involved ROS production that might be due to the occurrence of ferritinophagy. To this end, the autophagy inhibitor 3-MA was used to evaluate the contribution of autophagy in the regulation of apoptosis-related proteins. As shown in Figure 6(a), the action of PdtaA on the regulation of apoptosis-related proteins was significantly attenuated by addition of 3-MA. The quantification analysis of related proteins is shown in Figure 6(b). Similarly, the contribution of NCOA4 to apoptosis induction was also investigated. As expected, knockdown of NCOA4 neutralized the effect of PdtaA on regulation of Bax, Bcl-2, and caspase-8 (Figures 6(c) and 6(d)), supporting the hypothesis that PdtaA-induced apoptosis was associated with ferritinophagy.

### 2.6. PdtaA Induces Ferroptosis Associated with Ferritinophagy

**Figure 7:**
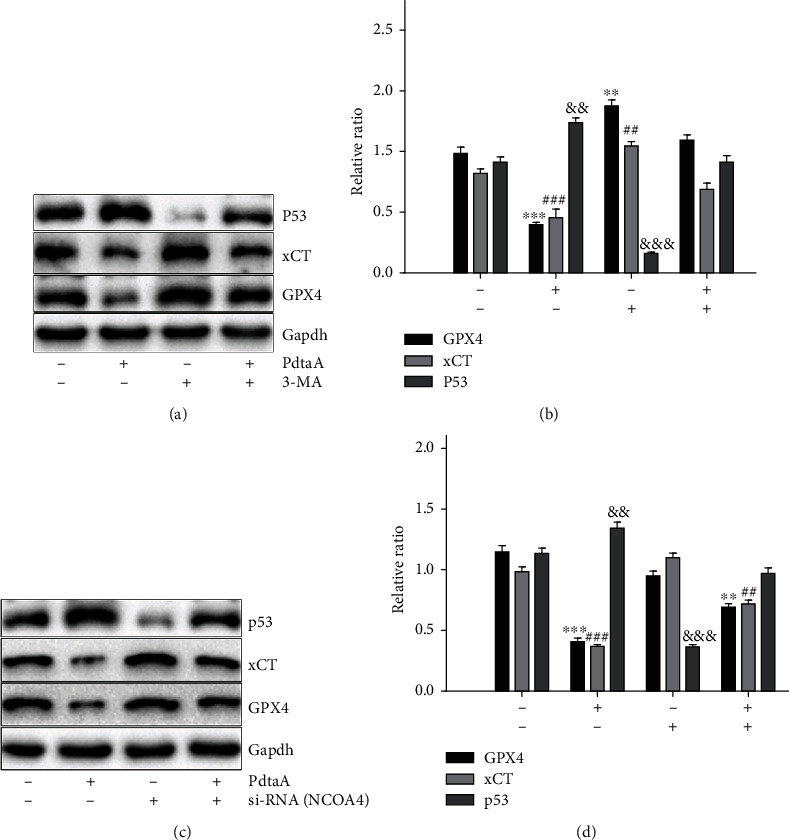
PdtaA induces ferroptosis associated with ferritinophagy. (a) 3-MA attenuated the effect of PdtaA on regulation of ferroptosis-related proteins and (b) quantification. (c) NCOA4 contributed to PdtaA-induced ferroptosis and (d) quantification. ^∗∗^^,&&,##^*P* < 0.05, ^∗∗∗^^,&&&,###^*P* < 0.01 vs. the control.

It has been shown that ferroptosis correlated with occurrence of ferritinophagy in different cell lines; therefore, it was considered if there was similar associated in action of PdtaA in HepG2 cells. To confirm this hypothesis, 3-MA was used to inhibit autophagy following PdtaA exposure in HepG2 cells. Western blotting analysis revealed that the action of PdtaA on ferroptosis induction was significantly attenuated by addition of 3-MA (Figures 7(a) and 7(b)), indicating that ferroptosis was a type of autophagy-dependent cell death, consistent with that reported previously. Similarly, the contribution of ferritinophagy to ferroptosis was investigated by knockdown of NCOA4. Figures 7(c) and 7(d) show that knockdown of NCOA4 resulted in attenuation of PdtaA-mediated regulation of GPx4, xCT, and p53, supporting the conclusion that PdtaA induced ferroptosis associated with the occurrence of ferritinophagy. In addition, the effects of 3-MA and NCOA4 knockdown on p53 were realized. This situation may originate from the facts that both 3-MA and knockdown of NCOA4 inhibited autophagy (or ferritinophagy), leading to repression of oxidative stress. As a stress responder, p53 downregulation seems to be a normal reaction in autophagy inhibition. Conversely, PdtaA treatment resulted in enhanced autophagy, accordingly p53 was upregulated.

## 3. Discussion

Chemotherapeutic agents induce the proliferation inhibition of cancer cells involving multiple pathways, such as apoptosis, cell cycle arrest, and autophagy. The cell cycle is the basis of cell proliferation and divided into different stages, and each stage has its own unique features [36]. The S phase is the stage of DNA synthesis and histone synthesis before cell division. However, DNA breakage will stop DNA replication and trigger DNA repair mechanisms that promote the phosphorylation of the CDK2/Cyclin E complex at the S phase detection site, which ultimately leads to prolongation of the S phase [37, 38]. Therefore, the downregulation of CDK2 would result in higher proportion in the S phase cells as observed in the present study (Figure 1(f)), which was consistent with that reported previously [39]. On the other hand, Ki-67 depletion also associated with the upregulation of p21 and involved cell cycle progression and the increased p21 also affected G2/M genes except G1/S [40]. In addition, p21 was also regulated by p53; therefore, alterations in those genes may contribute to the proliferation inhibition induced by PdtaA in the current study. ROS is widely involved in the action of chemotherapeutic agents. Similarly, PdtaA induced proliferation correlated with the ROS production (Figure 1(c)). NAC could neutralize the activity of ROS (Figure 1(d)); however, it did not fully rescue the proliferation inhibition and cell cycle arrest induced by PdtaA (Figures 1(b) and 1(f)). This implied that ROS production partly contributed to the proliferation inhibition.

Apoptosis is the most common form of cell death, initiated and executed by a unique family of cysteine-dependent aspartate-directed proteases. The extrinsic (death receptor-dependent) and the intrinsic (mitochondrial) pathways are two major well-studied apoptotic pathways [41]. The intrinsic pathway of apoptosis is tightly regulated by the ratio between two main groups of Bcl-2 family proteins: proapoptotic Bcl-2 proteins (including Bad, Bid, and Bax) and antiapoptotic Bcl-2 proteins (such as Bcl-2 itself, Bcl-xL, and myeloid cell leukemia 1 (Mcl-1) [42]. Western blotting analysis in the present study supported the hypothesis that apoptosis was involved because Bax and caspase-8 were upregulated, whereas Bcl-2 was downregulated after exposure of HepG2 cells to PdtaA (Figure 2). These results were similar to those reported previously [43]. In addition, ferroptosis is a recently defined form of regulated cell death [35], which includes (i) generation of ROS, (ii) depletion of GPx4, (iii) accumulation of lipid hydroperoxides (lipid ROS), and (iv) availability of iron [44]. It has been shown that ferroptosis overcomes some shortage of chemotherapeutic drug in apoptosis induction. Therefore, exploitation of ferroptosis in response to specific compounds is also an attractive strategy in cancer therapy, especially for apoptosis-resistant cancer cells. Mechanistically, iron metabolism and lipid peroxidation signaling play central roles in ferroptosis induction and the common inducers include erastin and RSL3 [45]. Furthermore, the xCT inhibitor sulfasalazine suppresses cellular detoxification and enhances the anticancer effect of cisplatin by increasing drug transport [46]. Notably, cisplatin and iron salophene complexes also induce ferroptosis [47, 48]; however, the ligands or iron chelator as a ferroptosis inducer was few. Generally, both membrane-permeable (ciclopirox olamine (CPX), 311 and 2,2′-bipyridil (2,2-BP) and membrane-impermeable (DFO) iron chelators prevent cells from undergoing ferroptosis [49]. In addition, inactivation of GPx4 could result in GSH depletion, thus promoting tumor-specific ferroptosis [50]. On the other hand, depressing cystine/glutamate antiporter system Xc- would achieve same goal [51]. In the current study, it was revealed that iron chelator (PdtaA) treatment resulted in depletion of GSH and increased of lipid peroxidation, concomitant to decreases of GPx4 and xCT, indicating that this type of iron chelator is a potent ferroptosis inducer (Figure 3). However, whether the iron chelator functions as an ROS scavenger to prevent ferroptosis or promotes ferroptosis depends on the ferrous complex dynamics, such as redox potential and reaction rate with hydrogen peroxide.

Since ROS production played a critical role in action of PdtaA in the present study, the characteristics and origin of ROS were further investigated. Generally, the production of superoxide, oxygen peroxide, and hydroxyl radicals occurs in mitochondria; however, these may be produced by agents (such as *β*-nicotinamide adenine dinucleotide 2′-phosphate reduced tetrasodium salt hydrate (NADPH) oxidase in particular cell types) [52]. In addition, hydroxyl radicals are produced from lysosomes or the labile iron pool (LIP). The Fenton reaction that involves Fe^2+^/Fe^3+^ transition is responsible for hydroxyl radical formation. However, which species respond to distinct biological activity was difficultly determined for ROS were a mixture of various species. However, several studies have indicated that the Fenton reaction is the main source of ROS in the occurrence of ferroptosis [53, 54]. Ferritinophagy is a process of ferritin degradation in lysosomes that requires NCOA4, resulting in the release of Fe (II), an increase of LIP, and generating ROS via the Fenton reaction [55, 56]. Downregulation of ferritin and upregulation of NCOA4 in the current study indicated that PdtaA induced ferritinophagy (Figures 4 and 5), which triggered apoptosis (Figure 6). In addition, it was suggested that intracellular iron accumulation and lipid peroxidation are two central biochemical events leading to ferroptosis. A few studies have revealed that ferroptosis involves ferritinophagy [57], in accordance with our observation (Figure 7); however, whether ferritinophagy inducers also act as a ferroptosis inducers was not clear. The present study provided an example of an iron chelator that induced both ferritinophagy and ferroptosis, further strengthening the association between the two cellular events because autophagy inhibition and knockdown of NCOA4 all counteracted the action of PdtaA on ferroptosis induction. It should be addressed that ferritinophagy-mediated ROS production played a crucial role in apoptosis and ferroptosis induction, because elimination of ROS production by autophagy inhibition or ROS scavenger function could significantly attenuate cellular events, including proliferation.

In conclusion, PdtaA induced apoptosis, cell cycle arrest, and ferroptosis associated with ferritinophagy-mediated ROS production. However, the use of more cell lines to support these conclusions is required in the future. In addition, ferritinophagy inducers may not result in ferroptosis for other iron chelators; therefore, this requires more investigation in the future.

## 4. Materials and Methods

### 4.1. Cell Lines, Reagents, and Antibodies

The HepG2 cell line was obtained from HonorGene; Changsha Aibiwei Biotechnology Co., Ltd. Ferrostatin-1, MTT, di-2-pyridylketone, 3-methyladenin (3-MA), RPMI-1640, and other chemicals were purchased from Sigma-Aldrich; Merck KGaA. GPx4, xCT (SLC7A11), vimentin, nuclear receptor coactivator-4 (NCOA4), and LC3 antibodies were obtained from ProteinTech Group, Inc. Antibodies against p53, caspase-8, GAPDH, Bax, and Bcl-2 were purchased from Wuhan Boster Biological Technology, Ltd. Antibodies targeting E-cadherin and secondary antibodies (fluorescence-labeled for immunofluorescence) were purchased from Cell Signaling Biotechnology, Inc. Ferritin antibody for immunofluorescence was obtained from Santa Cruz Biotechnology, Inc. NCOA4 antibody for immunofluorescence was purchased from Atlas Antibodies. Secondary antibodies for western blotting were obtained from EarthOx Life Sciences.

### 4.2. Preparation of PdtaA

PdtaA (chemical name generated by ACDLabs: 3-[({2-[(pyridin-2-yl)methylidene] hydrazinyl} carbonothioyl) sulfanyl] acetic acid) was prepared based on a described previous protocol [24]. The final product (PdtaA) was prepared by reaction of 2-pyridylhydrazone dithiocarbamate (1 mmol) with 2-bromo acetic acid in absolute ethanol (5 ml). The resulting yellow solid was filtered and washed with ethanol. Thin-layer chromatography tracing (ethyl acetate/petroleum ether = 3 : 1) showed that the reaction was complete. Following flash chromatography, the purity of the compound was 95%. Yield: 87%; mp: 135.8°C; composition: C_8_H_9_N_3_O_2_S_2_; ^1^HNMR (Bruker, D_6_-DMSO): 8.65 (*d*, *H*, *J* = 4 Hz), 8.29 (s, H), 7.94 (*m*, 2*H*, *J* = 4, 8 Hz), 7.48 (*m*, 1*H*, *J* = 8 Hz), 4.09 (s, 2H). ^13^CNMR: 198.95, 156.19, 152.04, 149.11, 148.86, 145.32, 123.82, 36.75. ESI-MS (microTOF-Q III, Bruker): m/z: 281.9757 (M+K, calcd: 281.9773).

### 4.3. Cytotoxicity (MTT) Assay

In total, 70% DMSO was used to prepare a 10 mM PdtaA store solution. When used, the store solution was diluted with 70% to the required concentration. The MTT assay was conducted as previously described [24]. Briefly, HepG2 (5 × 10^3^/well) cells in exponential phase were seeded into a 96-well plate and incubated until the cells adhered. Next, various concentrations of PdtaA were added and incubated for a further 48 h at 37°C in a humidified atmosphere of 5% CO_2_. Finally, 10 *μ*l MTT solution (5 mg/ml) was added to each well and incubated for 4 h. After carefully removing the cell culture, 100 *μ*l DMSO was added in each well to dissolve the formed formazan. The absorption was measured using a microplate reader (MK3; Thermo Fisher Scientific, Inc.) at 490 nm. Percent proliferation inhibition was calculated for each concentration and the assay was performed in triplicate.

### 4.4. ROS Detection

HepG2 cells (1 × 10^5^/well) were seeded into a 6-well plate and treated as described in the section of cytotoxicity assay. The cells were treated with PdtaA (15 or 25 *μ*M) for 24 h. Then, the cell culture was removed, following PBS washing and trypsin digestion; finally, the cells were resuspended in H_2_DCF-DA containing serum-free culture medium and incubated for 30 min (active oxygen detection kit, Beyotime Biotechnology). Next, after removing, the H_2_DCF-DA contained medium by centrifugation and washing with PBS; the intracellular ROS assay was performed on a flow cytometer (Cytoflex, Beckman Coulter, USA).

### 4.5. Cell Cycle Analysis

The HepG2 cells (1 × 10^5^/well) were seeded in a 6-well plate and incubated for 24 h at 37°C (5% CO_2_). After removing the medium, the fresh medium with various concentrations of PdtaA was added to the wells. Following 24 h of incubation, the cells were digested by trypsin and harvested by centrifugation. After washing with PBS, the cells were fixed in 70% ethanol and stored at -20°C. The ethanol was removed and cells washed with PBS; then, the cells were suspended in 0.5 ml PBS containing 50 *μ*g/ml PI and 100 *μ*g/ml RNase and stained for 30 min. For each sample, 10,000 events were collected, and the fluorescent signal intensity was recorded and analyzed using CytExpert (Cytoflex, Beckman Coulter, USA).

### 4.6. Flow Cytometry Analysis of Apoptosis

HepG2 cells that treated either with or without PdtaA (15 and 25 *μ*M PdtaA) for 36 h (or 24 h) were collected after PBS washing and trypsin digestion. Then, an Annexin V and propidium iodide kit (Dojindo Molecular Technologies, Inc.) was used as the company recommended. The stained cells were subjected to flow cytometry analysis (Cytoflex, Beckman Coulter, USA)).

### 4.7. Immunofluorescence Analysis

Immunofluorescence analysis was conducted as described previously [11]. Briefly, the HepG2 cells (1 × 10^5^/well) were first cultured in a 24-well plate with cover glass overnight. The cells were first fixed with 4% paraformaldehyde in PBS for 15 min at 37°C after PdtaA treatment for 24 h as aforementioned and then permeabilized with 0.2% triton-X-100 in PBS for 10 min. After blocking with 1% BSA in PBS for 30 min; the cells were incubated with either ferritin (H chain; Santa Cruz Biotechnology, Inc.) or combined with NCOA4 or LC3 (Altas Antibodies) primary antibodies based on the protocol recommended by the company, and the plate was shaken at 4°C for overnight. Then, removing the primary antibodies and washing with PBS, the cells were further incubated with fluorescence-labeled secondary antibody for 3 h at room temperature. Removing the secondary antibody, the cells were further counterstained with DAPI. Finally, a confocal laser scanning microscope (Nikon Eclipse Ts2; Nikon Corporation) was used to visualize the cells, and images of representative cells were captured.

### 4.8. Knockdown of NCOA4

The procedure for NCOA4-knockdown used small interfering (si-RNA-mate (siN0000001-1-5)) and siRNA (siG000008031A-1-5) (both Guangzhou RiboBio Co., Ltd.) as described previously [11]. Briefly, after removing culture and washing with PBS, HepG2 cells (1 × 10^6^) were transfected with 100 pmol of siRNA using Lipofectamine® Stem Transfection Reagent (Invitrogen; Thermo Fisher Scientific, Inc.) for 12 h based on a protocol recommended by the manufacturer. Next, PdtaA was added to the cells with complete medium for 24 h incubation at 37°C in a humidified atmosphere of 5% CO_2_. In addition, for immunofluorescence analysis, the HepG2 cells were first cultured in 24-well plate with cover glass overnight.

### 4.9. Western Blotting Analysis

As described previously [11], HepG2 cells treated with or without PdtaA were scraped off in lysis buffer (50 mM Tris-HCl, pH 8.0, 150 mM NaCl, 1.0% NP-40, 10% glycerol, and protease inhibitors) and hydrolyzed on ice for 30 min, followed by centrifugation at 14,000 x g. The clear supernatant was collected and stored at -80°C. The protein concentration was determined using a colorimetric Bio-Rad DC protein assay on a microplate reader MK3 at 570 nm. Proteins (30 *μ*g) were loaded on a 13% SDS-PAGE gel at 200 V for 1-2 h. Then, the separated proteins were subsequently transferred onto a PVDF membrane at 60 V for 1 h. The membrane was then blocked by 5% nonfat skimmed milk in TBS containing 0.1% Tween-20 for 2 h. Next, the membrane was incubated at 4°C overnight with the primary monoantibody at a dilution of 1 : 300 in the TBST buffer. Washed several times with TBST, the membrane was subsequently incubated with HRP-conjugated secondary antibody (1 : 2,000 in TBST) for 1 h at room temperature. The protein bands were detected using a supersensitive ECL solution (Boster Biological Technology) and visualized on an SYNGENE G: BOX Chemi XX9 (Syngene Europe). Quantifications of protein bands intensities were performed using ImageJ software (National Institutes of Health).

### 4.10. Statistical Analysis

Data were analyzed with using SPSS 19 (IBM Corp). Comparisons between multiple groups were performed using one-way ANOVA with Dunnett's post hoc correction. Comparison between two groups was performed using Student's *t*-tests. Results are presented as the mean ± SEM. *P* < 0.05 was considered to indicate a statistically significant difference.

## Data Availability

All data generated or analyzed during this study are included in this published article.

## Supplementary Materials

Supplementary Materials Figure S1: 2-Pyridylhydrazone dithiocarbamate s-acetic acid induces proliferation inhibition associated with downregulation of Ki-67. (A) Western blotting analysis and (B) quantification analysis from (A). ^∗∗^*P* < 0.05 and ^∗∗∗^*P* < 0.01 vs. the control. Figure S2: PdtaA induces cell cycle arrest associated with feritinophagy. (A) Western blotting analysis and (B) quantification from (A). ^##^*P* < 0.05, ^∗∗∗^*P* < 0.01, and ^^^^^*P* < 0.01 vs. the control. Figure S3: addition of NAC or 3-MA individually attenuated the ability of PdtaA in apoptosis induction. Figure S4: PdtaA treatment resulted in alteration in both GSH and lipid peroxidation. Changes of (A) GSH and (B) lipid peroxidation at different conditions. ^∗∗^*P* < 0.05 and ^∗∗∗^*P* < 0.01 vs. the control (DMSO). Figure S5: effect of a ferroptosis inhibitor on PdtaA-mediated regulation of xCT, GPx4, and p53. (A) Western blotting analysis and (B) quantification. ^∗∗^*P* < 0.05, ^^^^*P* < 0.05, ^&&^*P* < 0.05, and ^^^^^*P* < 0.01 vs. the control (DMSO). Figure S6: alterations in NCOA4 fluorescence between siRNA-mate and siRNA-NCOA4 treatments. The HepG2 cells treated with either siRNA-mate (A) or siRNA-NCOA4 (B); and (C) quantification analysis from (A) and (B). Nuclei were stained by DAPI in blue and NCOA4 was stained in green. ^∗∗^*P* < 0.05 vs. the control. Figure S7: alteration of iron abundance when the cells are subjected to PdtaA treatment or siRNA. ^∗∗^*P* < 0.05 and ^∗∗∗^*P* < 0.01 vs. the control (DMSO).
