# Supplementary material for: Ferritinophagy-Mediated ROS Production Contributed to Proliferation Inhibition, Apoptosis, and Ferroptosis Induction in Action of Mechanism of 2-Pyridylhydrazone Dithiocarbamate Acetate
Source: Oxid Med Cell Longev. 2021 Oct 14;2021:5594059. doi: 10.1155/2021/5594059 (PMC8531783; doi:10.1155/2021/5594059)
Supplement: Supplementary Materials — Figure S1: 2-Pyridylhydrazone dithiocarbamate s-acetic acid induces proliferation inhibition associated with downregulation of Ki-67. (A) Western blotting analysis and (B) quantification analysis from (A). ∗∗P < 0.05 and ∗∗∗P < 0.01 vs. the control. Figure S2: PdtaA induces cell cycle arrest associated with feritinophagy. (A) Western blotting analysis and (B) quantification from (A). ##P < 0.05, ∗∗∗P < 0.01, and ^^^P < 0.01 vs. the control. Figure S3: addition of NAC or 3-MA individually attenuated the ability of PdtaA in apoptosis induction. Figure S4: PdtaA treatment resulted in alteration in both GSH and lipid peroxidation. Changes of (A) GSH and (B) lipid peroxidation at different conditions. ∗∗P < 0.05 and ∗∗∗P < 0.01 vs. the control (DMSO). Figure S5: effect of a ferroptosis inhibitor on PdtaA-mediated regulation of xCT, GPx4, and p53. (A) Western blotting analysis and (B) quantification. ∗∗P < 0.05, ^^P < 0.05, &&P < 0.05, and ^^^P < 0.01 vs. the control (DMSO). Figure S6: alterations in NCOA4 fluorescence between siRNA-mate and siRNA-NCOA4 treatments. The HepG2 cells treated with either siRNA-mate (A) or siRNA-NCOA4 (B); and (C) quantification analysis from (A) and (B). Nuclei were stained by DAPI in blue and NCOA4 was stained in green. ∗∗P < 0.05 vs. the control. Figure S7: alteration of iron abundance when the cells are subjected to PdtaA treatment or siRNA. ∗∗P < 0.05 and ∗∗∗P < 0.01 vs. the control (DMSO). [file 5594059.f1.docx]

**Ferritinophagy-mediated ROS production contributed to proliferation inhibition, apoptosis and ferroptosis induction in action of mechanism of 2-pyridylhydrazone dithiocarbamate acetate**

Longlong Li^1#^, Hao Li^1#^, Yongli Li^2#^, Jiankang Feng^3^, Deng Guan^3^, Yalei Zhang^3^, Yun Fu^3^, Shaoshan Li^1*^ and Changzheng Li^3,4*^

^1^Department of Surgery, The Third Affiliated Hospital of Xinxiang Medical University; ^2^Department of Histology and Embryology, Sanquan College of Xinxiang Medical University; ^3^College of Basic Medical Science, Xinxiang Medical University; ^4^Experimental Teaching Center of Biology and Basic Medical Sciences, Sanquan College of Xinxiang Medical University, Xinxiang, Henan 453003, P. R. China

**Supplementary Materials**

**PdtaA induces proliferation inhibition associated with downregulation of Ki-67**

Ki-67 is known to be a cell proliferation marker and also a marker for a cell response to drugs that target cell proliferation. Therefore, it was hypothesized that PdtaA-induced proliferation inhibition might be associated with the regulation of Ki-67. To this end, western blotting analysis was conducted, as showed in Fig. S1. PdtaA could inhibit the expression of Ki-67 (Fig. S1A) and the significant alterations were observed (Fig. S1B; P<0.05 and P<0.01). This indicated that PdtaA-induced proliferation inhibition was associated with downregulation of Ki-67.


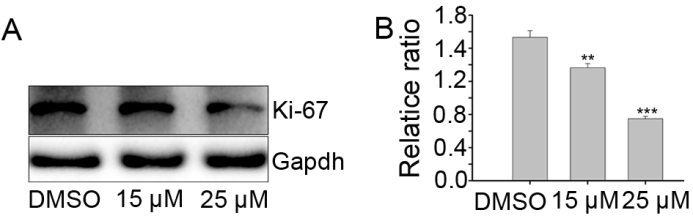


**Figure S1**. 2-pyridylhydrazone dithiocarbamate s-acetic acid induces proliferation inhibition associated with downregulation of Ki-67. (A) Western blotting analysis and (B) quantification analysis from (A). ^**^P<0.05 and ^***^P<0.01 vs control.

**PdtaA induces cell cycle arrest associated with feritinophagy**

PdtaA-induced cell cycle arrest was ROS-dependent, thus the origin of ROS production was further analyzed. In view of the previous observation that dithiocarbamate derivatives could induce ferritin degradation in lysosomes [1], it was considered whether PdtaA might have similar function. To this end, the level of ferritin, NCOA4 and cell cycle-related proteins was determined. As shown in Fig. S2, PdtaA could induce a downregulation of ferritin and upregulation of NCOA4, indicating an occurrence of ferritinophagy (Fig. S2A). Meanwhile, CDK2 levels also changed with the enhanced ferritinophagy (Fig. S2A), indicating there was an association between CDK and ferritinophagy. The term ferritinophagic flux, defined as NCOA4/ferritin, could be used to describe this association. Fig. S2B showed that PdtaA caused an increase of ferritnophagic flux, which led to downregulation of CDK2. This indicated that CDK2 triggered cell cycle arrest associated with an increase of ferritinophagic flux [1].

**
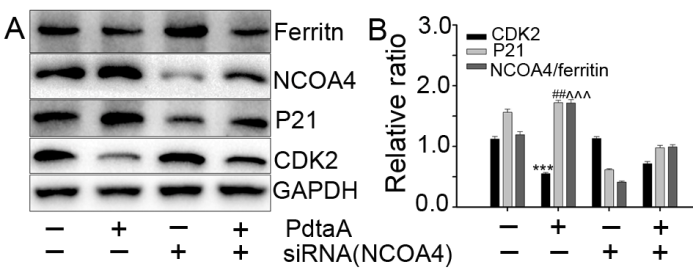
**

**Figure S2**. PdtaA induces cell cycle arrest associated with feritinophagy. (A) Western blotting analysis and (B) quantification from (A). ^##^P<0.05, ^***^P<0.01 and ^^^^^P<0.01 vs control.

**ROS production and autophagy contributed to PdtaA induced apoptosis**

As shown in Fig. 2, PdtaA treatment resulted in significant apoptosis, which made contribution to the proliferation inhibition. In addition, it is well documented that ROS are causal factor for apoptosis induction, whether the apoptosis induction involved ROS production was further determined. To this end, the cells were pre-treated by NAC. As expected, PdtaA treatment (24 h) resulted in 22.80% cells apoptosis (Fig. S3), however, the addition of NAC attenuated the action of PdtaA on apoptosis induction (from 22.80 % to 13.26%), supporting that the apoptosis induction involved ROS production. Furthermore, ROS production also caused autophagy occurrence, therefore, an autophagy inhibitor, 3-MA was used to determine whether autophagy involved the action of the agent in mechanism. Clearly, 3-MA addition indeed weaken the regulatory effect of PdtaA on apoptosis induction (from 22.80% to 12.5%), supporting that autophagy was also involved in action of PdtaA.

**
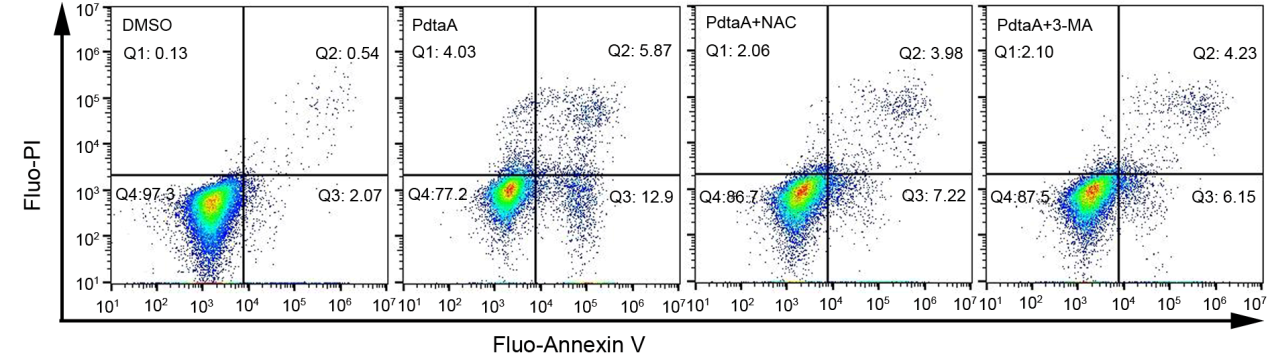
**

**Figure S3**. Addition of NAC or 3-MA individually attenuated the ability of PdtaA in apoptosis induction.

**Depletion of reduced GSH and increased lipid peroxidation is associated with ferroptosis**

The GSH assay was performed based on its reaction with 5,5’-dithiobis-(2-nitrobenzoic acid) (DTNB). The protocol was followed as the company recommended (Nanjing Jiancheng Bioengineering Institute). Briefly, the cells either treated with PdtaA or without treatment were collected and lysed in an isosmotic solution. The lysate was further treated further and the precipitate was centrifuged. Next, 100 μl of cellular supernatant was mixed with 25 μl DTNB and 100 μl buffer, and the mixture was incubated for 5 min at room temperature. The absorbance at 405 nm that correlates GSH concentration was measured with an ELISA reader (Benchmark Plus Reader; Bio-Rad Laboratories, Inc.). Lipid peroxidation analysis was performed based on spectrophotometry, in which the ferrous ion is oxidized by lipid hydroperoxides to the ferric ion and subsequently reacts with thiocyanate to form a colored complex [2]. The assay was performed according to the previously described protocol [3]. Briefly, the trypsinized cells were collected and treated with the PdtaA for 24 h. The supernatant was removed by centrifugation and washed with PBS. The peroxidized lipids were extracted using deoxygenated CHCl_3_/MeOH (2:1, v/v mixture; 1,000 μl), and the lipids were transferred to a 5 ml volumetric flask, which contained 100 μl of ferrous sulfate (0.2 M HCl) and 100 μl of 3% deoxygenated thiocyanate (methanol) for 60 min. Finally, deoxygenated CHCl_3_/MeOH solvents were added to the given volume. The absorbance at 500 nm was measured using a UV-2450 spectrophotometer (Shimadzu Corporation). The molar absorptivity of the ferric thiocyanate complex expressed per mol of LOOH was determined to be 58,440 M^-1^ cm^-1^ [2].

As shown in Fig. S4A, the abundance of reduced GSH was significantly decreased upon PdtaA treatment, in accordance with increase of ROS production (Fig. 1). The addition of 3-MA markedly attenuated the action of PdtaA, indicating that autophagy was involved in the process. Similarly, inhibition of p53 attenuated the action of PdtaA on regulation of GSH. Since GSH decreased, the lipid peroxidation would occur. As expected, the level of peroxidized lipids was significantly increased (Fig. S4B) and addition of 3-MA attenuated the peroxidation of lipids. This supported the hypothesis that PdtaA-induced ROS production involved autophagy. Notably, the ferroptosis inhibitor (0.5 µM) partly neutralized the increase in lipid peroxidation. These data suggested an occurrence of ferroptosis involved in action of PdtaA.


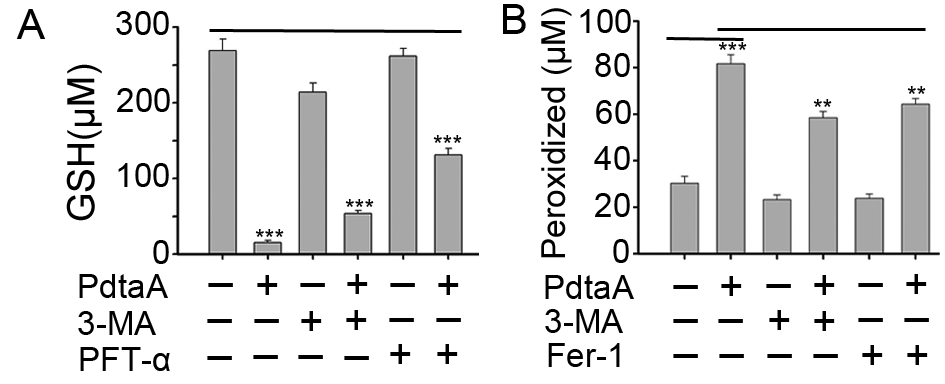


**Figure S4**. PdtaA treatment resulted in alteration in both GSH and lipid peroxidation. Changes of (A) GSH and (B) lipid peroxidation at different conditions. ^**^P<0.05 and ^***^P<0.01 vs control (DMSO).

Additional evidence from western blotting analysis also supported this conclusion, because ferroptosis inhibitor ferrostatin-1 could partly neutralize the action of PdtaA on regulation of xCT and GPx4 (Fig. S5).


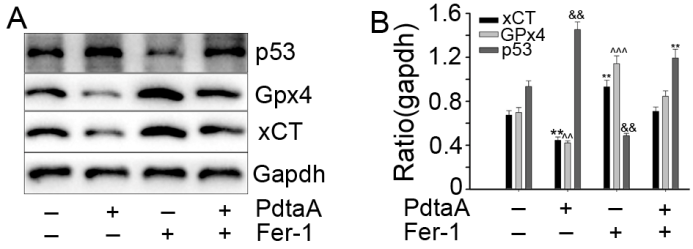


**Figure S5**. Effect of a ferroptosis inhibitor on PdtaA-mediated regulation of xCT, GPx4 and p53. (A) Western blotting analysis and (B) quantification. ^**^P<0.05, ^^^^P<0.05, ^&&^P<0.05, ^^^^^P<0.01 vs control (DMSO).

**Knockdown of NCOA4 by siRNA**

To determine the efficiency of knockdown of NCOA4, the immunofluorescence analysis was conducted. The photos of labeled NCOA4 in control (siRNA-mate) and siRNA-NCOA4 under fluorescence confocal microscope were recorded (Fig. S6A-B), the average fluorescence intensity from 6 cells was obtained by using imageJ, the results were presented in Fig. S6C. Clearly, the NCOA4 was significantly downregulated by the siRNA.


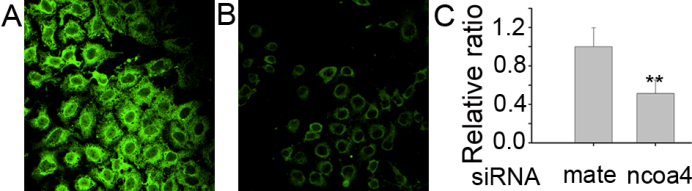


**Figure S6**. Alterations in NCOA4 fluorescence between siRNA-mate and siRNA-NCOA4 treatments. The HepG2 cells treated with either siRNA-mate (A) or siRNA-NCOA4 (B); and (C) quantification analysis from (A) and (B). Nuclei were stained by DAPI in blue and NCOA4 was stained in green. **P<0.05 vs control.

**PdtaA induces decreased total iron abundance that is relative to autophagy**

The total iron content of HepG2 cells was determined by atomic absorption spectroscopy (AAS) (Persee, Beijing, China, model TAS-900) based on a method reported previously with some modification [4]. The downregulation of NCOA4 was achieved by siRNA as described previously [1]. The cultured cells were washed three times with a HEPES-buffered solution (154 µM NaCl, 10 mM HEPES, pH 7.4). Cells were lysed by addition of HNO_3_ with heating, and the iron content was determined by AAS. A cell-free sample was prepared in same way and used as a blank. The results are presented in Fig. S7. PdtaA treatment resulted in a significant decrease of abundance of iron (total iron), implying downregulation of ferritin; while knockdown NCOA4 led to obvious an increase in iron abundance; however, knockdown NCOA4 significantly attenuated the decrease in iron abundance induced by PdtaA, those supported that PdtaA induced an occurrence of ferritinophagy.


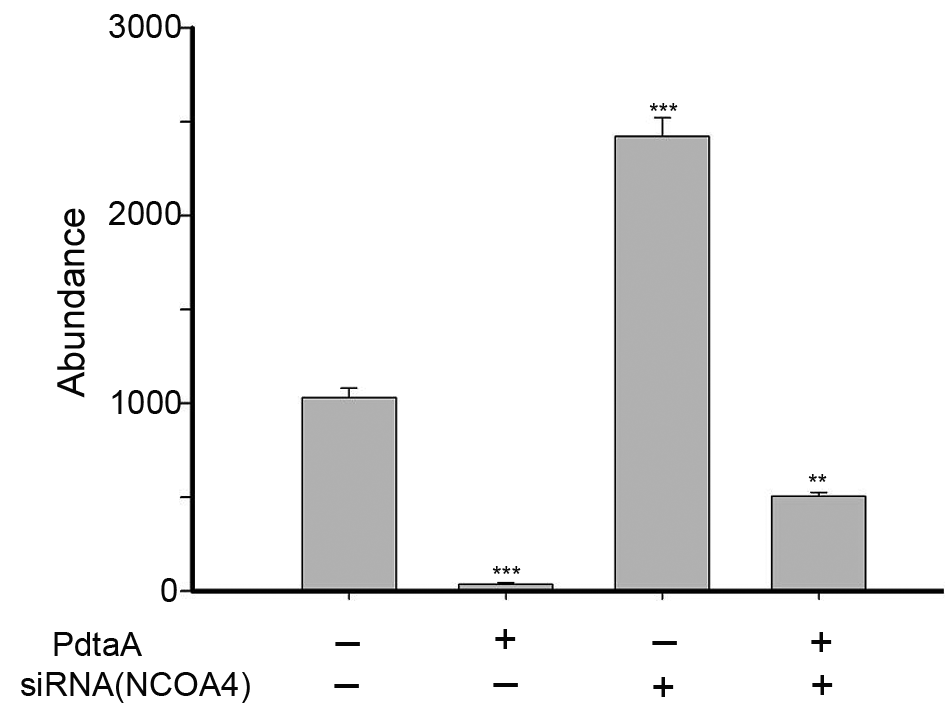


**Figure S7**. Alteration of iron abundance when the cells are subjected to PdtaA treatment or siRNA. **P<0.05 and ***P<0.01 vs control (DMSO).

**References**

1. Y.J. Sun, C.P. Li, J.K. Feng, et al., “Ferritinophagic flux activation in CT26 cells contributed to EMT inhibition induced by a novel iron chelator, PdtaA,” Oxidative Medicine and Cellular Longevity, vol. 2019, article ID 8753413, 2019.
2. A.C. Gasparovic, M. Jaganjac, B. Mihaljevic, et al., “Assays for the measurement of lipid peroxidation,” Methods in Molecular Biology, vol. 965, pp. 283-296, 2013.
3. T.F. Huang, Y.J. Sun, Y.L. Li, et al., “Growth inhibition of a novel iron chelator, DpdtC, against hepatoma carcinoma cell lines partly attributed to ferritinophagy-mediated lysosomal ROS generation,” Oxidative Medicine and Cellular Longevity, vol 2018, article ID 4928703, 2019.
4. J. Riemer, H.H. Hoepken, H. Czerwinsk, et al., “Colorimetric ferrozine-based assay for the quantitation of iron in cultured cells,” Analytical Biochemistry, vol. 331, no. 2, pp. 370-375, 2004.
